# Symptom Flares in Endometriosis: Burden, Self‐Management and Barriers to Care in a Cross‐Sectional Survey

**DOI:** 10.1111/1471-0528.70211

**Published:** 2026-03-13

**Authors:** Lydia Coxon, Emma Evans, Francine Toye, Emma Cox, Katy Vincent

**Affiliations:** ^1^ Nuffield Department of Women's & Reproductive Health University of Oxford Oxford UK; ^2^ Oxford University Hospitals NHS Foundation Trust Oxford UK; ^3^ Endometriosis UK

**Keywords:** chronic pelvic pain, endometriosis, flares, pain

## Abstract

**Objective:**

To explore the characteristics of symptom flares, individual experiences and behaviours during flares in people with endometriosis.

**Design:**

Online questionnaire shared on patient support sites.

**Setting:**

People with a confirmed or working diagnosis of endometriosis (a working diagnosis is given by clinicians based on symptoms/history; individuals may or may not go on to have further imaging/surgical investigations).

**Population or Sample:**

A total of 236 responses were collected.

**Methods:**

Descriptive and comparative analysis of quantitative data, and thematic analysis of qualitative data.

**Main Outcome Measures:**

The characteristics, triggers, treatments and strategies for symptom flares together with perceived predictability and self‐efficacy in relation to flares, healthcare access during flare, advice received and overall endometriosis‐related quality‐of‐life.

**Results:**

We identified a wide variation in the characteristics and treatments/strategies. 31.2% stated that they were ‘not at all’ confident coping with long flares, and around 1/3 of participants found flares ‘not at all’ predictable. Only 35.3% reported receiving advice from a healthcare provider about flares. We developed 5 themes to suggest why participants did not contact healthcare providers: ‘what can they do?’, ‘I can cope, it will end’, ‘broken healthcare system’, ‘perceived dismissal and gaslighting’ and ‘symptoms stop me’.

**Conclusions:**

Flares have a large impact on quality‐of‐life and are clinically very important. Individuals do not commonly receive advice from healthcare providers or contact healthcare providers during a flare. More research, in a more diverse sample, is needed to identify mechanisms underlying flares, as well as developing and disseminating management tools to prevent, manage and treat flares.

## 1 Introduction

Endometriosis is a chronic condition, characterised by finding tissue resembling the uterine lining (endometrium) outside the uterus [1]. It is estimated to affect 1 in 10 reproductive age people assigned female at birth [1]. Pelvic pain symptoms are common in people with endometriosis, including dysmenorrhoea, dyspareunia, non‐cyclical pain, dysuria and dyschezia [2].

Whilst there has in recent years been greater awareness of, and research into, endometriosis‐associated pain (EAP) [2, 3], one area that has remained under‐explored is symptom flares. People with endometriosis often report, both clinically and on social media, that they experience large fluctuations in their symptoms, including *flares* where their symptoms are much worse than usual. Currently there are no treatments aimed at stopping or reducing the frequency and severity of these flares [4]. As a result, flares can result in hospital admissions, with large associated costs to the individual, healthcare provider and society [5].

We have previously explored symptom flares in a cohort of people with chronic pelvic pain (CPP) [6], including a subgroup with surgically confirmed endometriosis. It was clear from this study that symptom flares are common and bothersome, and that they are associated with lower quality‐of‐life. Importantly, the total amount of time in flare correlated with both anxiety and helplessness. Thus, addressing flares through prevention, treatment and/or improved management could potentially lead to marked benefit for a large proportion of those living with CPP.

The overarching objective of this study was therefore to further our understanding of symptom flares in those with EAP. Specifically, we wanted to understand where people receive information regarding flares from, and how helpful this is. We also wanted to find out which treatments and strategies that individuals had tried to prevent and/or treat flares and their effectiveness. As flare management is not described in current clinical guidance for endometriosis [7, 8], it is likely that many people get their information from social media/support groups rather than from healthcare providers. Given the challenges of unpredictable events [9, 10, 11] and the increasing evidence supporting the importance of pain self‐efficacy [12], we were interested to explore whether the predictability of flares and flare self‐efficacy related to quality‐of‐life. Should these relationships exist, this would be a framework on which to develop a flare management intervention for those with EAP.

## 2 Methods

### 2.1 Questionnaire Design

The questionnaire used in this study (see Supporting Information) combined several validated tools with additional questions to collect other information of interest where validated tools were not available. Items were not randomised.

We used the World Endometriosis Research Foundation's Endometriosis Phenome and Biobanking Harmonisation Project (EPHect) clinical covariates tool to collect demographic and clinical data [13]. Pelvic pain symptoms were assessed as *having ever been present*: if present, the duration in years and average and worst pain intensity were recorded, using a numerical rating scale (0 = no pain: 10 = worst pain imaginable). Quality of life was assessed with the Endometriosis Health Profile (EHP‐30) [14, 15].

Information about flares was assessed with a modified version of the Multidisciplinary Approach to the Study of Chronic Pelvic Pain (MAPP) consortium Flares Questionnaire [16, 17, 18, 19]. As this questionnaire was designed for urological pelvic pain, we had previously modified it to widen its applicability to other types of pelvic pain (including endometriosis‐associated pain) [6]. The questionnaire defines flares as ‘symptoms that are much worse than usual’, broken down into short (< 1 h), medium (> 1 h to < 1 day) and long (> 1 day) flares. It asks about: duration of flares; frequency of flares; the severity of symptoms (0 = no symptoms: 10 = worst symptoms); response to flare (e.g., healthcare utilisation and medication); most bothersome symptoms; impact on activities; how much they thought about symptoms during flares; symptom ‘bother’; things that triggered flares. Flare self‐efficacy (i.e., confidence to undertake activities during a flare) and perceived predictability of flares were also assessed using a 0–5 scale (0 = not at all predictable/confident: 5 = entirely predictable/completely confident). Data on strategies (including medication) used to prevent/treat flares, and effectiveness were collected (yes/no) with free text boxes to report additional information. The effectiveness was scored from −10 to +10 (with 0 = no change, +10 = symptoms went away completely, −10 = symptoms became very much worse). We also collected data on where they received advice from (e.g., healthcare providers/support groups/social media) and how helpful this was (‘not at all’, ‘only a little’, ‘some’, ‘a lot’), with free text boxes to include any further information. Adaptive questioning/branching was used to reduce burden and complexity.

Co‐investigator EC (CEO of Endometriosis UK) reviewed and edited the initial version of the questionnaire to ensure understandability and acceptability. The questionnaire was then piloted in those with lived experience, and their feedback was incorporated into the design of the final version.

### 2.2 Details of Ethics Approvals

This study was approved by the Medical Sciences Interdivisional Research Ethics Committee, University of Oxford, R91127/RE001. Implied consent was attained via tick boxes to ensure the participant was aged 18 or over and that they agreed to take part in the study.

### 2.3 Recruitment & Data Collection

The advert for the study was shared by support groups (Endometriosis UK, Endometriosis Foundation; Endometriosis Association of Ireland; Endometriosis SHE Trust UK; endometriosis.org) and on sites including the Nuffield Department of Women's and Reproductive Health website. Whilst we predominantly aimed to capture UK responses, interested participants from outside the UK were able to complete the survey if they became aware of it. The survey was voluntary and advertised as exploring the symptoms of endometriosis flares and was an open survey (convenience sample).

The survey was hosted on Jisc Online Surveys v3 and was open to responses from 5/3/2024 to 30/10/2024. Participants were excluded from analysis if they did not report a diagnosis of endometriosis (‘Yes, I have a working diagnosis, awaiting tests’; ‘Yes, I have a working diagnosis, I am not awaiting tests’; ‘Yes, by imaging (MRI or ultrasound)’; ‘Yes, by laparoscopy’). Participation in the survey was anonymous, and we did not ask for any data that could directly identify the individual. Cookies were not used and we could not access information on IP address. Participants were not allowed to partially complete the survey and return later, although they could go back to previous questions. No incentives were provided for participation. Only completed surveys are collected and analysed.

### 2.4 Analysis

All statistical analysis was carried out on SPSS v29 with figures created on GraphPad Prism v10 and wordclouds.co.uk.

Reported are medians, (interquartile ranges (IQR)), [ranges], {95% confidence intervals (CIs)} for the median, with IQR calculated using weighted average and CIs with bootstrapping based on 1000 bootstrap samples. For correlations, Spearman's correlation was used. For comparisons of helpfulness of advice received from healthcare providers and social media/support groups, the Wilcoxon Signed Ranks test was used. All *p* values were corrected using Bonferroni correction.

For frequency of flares, all are reported as per month, calculated as 4.35 weeks per month and 30.42 days per month, with medians taken if ranges given. To calculate the total time in ‘flare’ (total hours per month in flare), for each individual we multiplied the reported average duration of flare (hours) by the reported frequency of flare (per month) for each length flare (short, medium and long) and then summed these together.

Whilst the survey was predominantly quantitative in nature, we did collect qualitative data on a limited number of questions, primarily around contacting health professionals. Free text describing reasons for not contacting a health professional was coded inductively and analysed thematically by two researchers (LC and FT) [20]. Three researchers (LC, FT and KV) discussed each theme and its free text data to bring a range of perspective and to ensure that valuable nuance was not lost. LC is a researcher, with a background in neuroscience without any clinical experience; FT is an anthropologist and physiotherapist; KV is a practising gynaecologist with a research background in pain science.

## 3 Results

### 3.1 Demographics

Two hundred thirty‐six responses meet inclusion criteria, with a median age of 31 (IQR 26–37), the majority located in the UK and with average pelvic pain intensity NRS scores of median 7 for dysmenorrhoea and dyspareunia and 8 for non‐cyclical pain. Further age, location, pain prevalence, pain intensity ratings, duration of pain and EHP‐30 subscale scores can be seen in Table 1.

**TABLE 1:** Demographics data on age, pain intensity ratings, pain duration and Endometriosis Health Profile (EHP‐3) subscales as well as prevalence of pain types and location.

	N	%	Median	IQR	Range	95% CIs
Age	228		31	26–37	18–57	30–33
Diagnosis						
Working diagnosis awaiting tests	18	7.6				
Working diagnosis, not awaiting tests	9	3.8				
Imaging diagnosis	67	28.4				
Laparoscopy diagnosis	170	72.0				
Location						
England	183	77.9				
Wales	7	3				
Scotland	16	6.8				
Northern Ireland	1	0.4				
Outside the UK	28	11.9				
Dysmenorrhoea						
None	0	0				
Mild	6	2.6				
Moderate	29	12.3				
Severe	200	85.1				
Dysmenorrhoea Worst	235		9	8–10	0–10	9–9
Dysmenorrhoea Average	235		7	6–8	0–10	7–7
Dyspareunia						
Ever experienced	189	80.4				
Not experienced	23	9.8				
Not had vaginal sexual intercourse/penetration	14	6				
Prefer not to say	9	3.8				
Dyspareunia Worst	188		7	5–8	0–10	6–7
Dyspareunia Average	187		5	3–7	0–10	5–6
Non‐cyclical pain						
Ever experienced	211	90.2				
Not experienced	23	9.8				
Non‐cyclical pain Worst	211		8	7–9	0–10	8–8
Non‐cyclical pain Average	211		6	5–8	0–10	6–7
Duration of pelvic pain	184		14	10–20	0.01–42	12–15
EHP‐30						
EHP‐30 Pain	230		63.6	50–73.3	0–100	59.1–65.9
EHP‐30 Control & Powerlessness	234		83.3	62.5–95.8	0–100	79.2–87.5
EHP‐30 Emotional Wellbeing	228		58.3	41.7–70.8	0–100	54.2–58.3
EHP‐30 Social Support	233		75	50–87.5	0–100	68.8–75
EHP‐30 Self Image	233		75	58.3–91.7	0–100	75–75
EHP‐30 Work Life	179		60	25–62.5	0–100	37.5–62.5
EHP‐30 Relationship with children	53		50	25–62.5	0–100	37.5–62.5
EHP‐30 Sexual Intercourse	164		67.	40–85	0–100	60–75
EHP‐30 Medical Profession	173		62.5	43.8–81.3	0–100	56.3–68.8
EHP‐30 Treatment	166		66.7	50–75	0–100	62.5–75
EHP‐30 Infertility	89		75	56.3–100	0–100	68.8–87.5

*Note:* Shown are median, 95% confidence intervals (CIs), interquartile range (IQR) and range or number and percentage (%) as appropriate. Pain ratings were collected using Numerical Rating Scales 0–10. Duration of pelvic pain is given in years. EHP‐30 subscales were calculated according to user manual. EHP‐30 subscales give values from 0 to 100, where higher scores indicate worse quality‐of‐life. For EHP‐30 subscales, not relevant options given: for Work Life *n* = 70 not in paid work; for Relationship with children *n* = 183 do not have children; for medical profession *n* = 64 not relevant for them; for Treatment *n* = 65 not relevant; and for Infertility *n* = 152 not relevant for them. For diagnosis, participants could select multiple options (e.g., laparoscopy and imaging).

### 3.2 Characteristics of Flares

Of the cohort, 98% reported experiencing flares of at least one length, with 19% reporting experiencing flares of all lengths. The most common flare length was long flares (85.6%), with a median of 3 days duration (Table 2). Short and medium flares had a prevalence of 37.7% and 56.4% respectively. There were a range of pain and non‐pain symptoms associated with flares (Table 2, Table S1).

**TABLE 2:** Characteristics and burden of symptoms flares.

	Short	Medium	Long
**Prevalence** count *n* (%)	89 (37.7)	133 (56.4)	202 (85.6)
**Duration** Median (IQR) [range] {95% CIs}	30 min (20–45) [3–61] {30–30}	5 h (3–6) [0.5–24] {4–5.5}	3 days (2.81–7) [1–25] {3–4}
**Frequency** per month Median (IQR) [range] {95% CIs}	8.7/twice per week (2–68.445) [0.08–304.20] {4.35–17.4}	4.35/once per week (2–13.05) [0.025–152.1] {3–6.5}	1.5 (1–3) [0.08–17.4] {1.3375–2}
**Symptoms during flare (NRS)**			
Pain, pressure and discomfort associated with your pelvis and/or bladder	7 (6–9) [0–10] {7–8}	8 (6–10) [2 – 10] {8–8}	8 (7–10) [2 – 10] {8–8}
Urgency to urinate	5 (2–8) [0–10] {4–6}	5 (1–7) [0–10] {4–6}	5 (3–8) [0–10] {5–6}
Urgency to open bowels	6 (2–8) [0–10] {4–6}	5 (2–7.75) [0–10] {4–6}	6 (3–8) [0–10] {5–6}
Frequency of urinating	5 (2–8) [0–10] {4–6}	5 (1–7) [0–10] {4–6}	5 (2–8) [0–10] {5–6}
Frequency of pooing	4 (1–6) [0–10] {3–5}	4.5 (1–6) [0–10] {3.51–5}	5 (2–7) [0–10] {3–8}
Overall bladder or pelvic pain symptoms	7 (5–9) [0–10] {7–8}	8 (5.25–9) [0–10] {7–8}	8 (6–10) [0–10] {8–8}
Overall pain symptoms that were not bladder or pelvic	7 (4–8) [0–10] {6–7}	6 (4–8) [0–10] {6–7}	7 (5–8.5) [0–10] {7–7}
**Activities during flare, *n* (%)**			
Contact HCP	5 (5.618)	8 (6.015)	24 (11.881)
Go to see HCP	2 (2.247)	2 (1.504)	12 (5.941)
Go to A&E	4 (4.494)	7 (5.263)	20 (9.010)
Have medication changed	6 (6.742)	10 (7.519)	21 (10.396)
Rest	71 (79.775)	101 (75.940)	161 (79.703)
Take mediation used specifically to treat the flare	51 (57.303)	87 (65.414)	127 (62.871)
Other	11 (12.360)	9 (6.767)	32 (15.842)
**Single most bothersome symptom during flare, *n* (%)**			
Pain, pressure or discomfort in your bladder	3 (3.371)	8 (6.015)	8 (3.960)
Pain, pressure or discomfort in your vagina	12 (13.483)	4 (3.008)	14 (6.931)
Pain, pressure or discomfort on the skin around the entrance to the vagina and/or back passage	6 (6.742)	3 (2.256)	8 (3.960)
Pain, pressure or discomfort elsewhere in your pelvis	46 (51.685)	85 (63.910)	125 (61.881)
Pain or discomfort during or after sexual activity	3 (3.371)	3 (2.256)	1 (0.495)
Strong need to urinate with little or no warning	0 (0)	0 (0)	0 (0)
Strong need to open your bowels with little or no warning	5 (5.618)	4 (3.008)	5 (2.475)
Frequent urination during the day	0 (0)	0 (0)	0 (0)
Frequent urination at night	1 (1.124)	0 (0)	0 (0)
Frequent bowel opening during the day	0 (0)	1 (0.752)	1 (0.495)
Frequent bowel opening at night	0 (0)	0 (0)	0 (0)
Sense of not emptying your bladder completely	1 (1.124)	1 (0.752)	1 (0.495)
Sense of not emptying your bowels completely	1 (1.124)	2 (1.504)	3 (1.485)
Increased vaginal discharge	0 (0)	0 (0)	0 (0)
A feeling of bloating in the abdomen	4 (4.494)	11 (8.271)	12 (5.941)
Other	3 (3.371)	5 (3.759)	20 (9.901)
**Burden, *n* (%)**			
Keep from doing things you would usually do	78 (90.698)	115 (92)	194 (97.487)
Think about your symptoms	84 (97.674)	122 (96.825)	198 (99.497)
Bother	82 (95.349)	123 (96.850)	197 (98.994)
**Flare self‐efficacy**	2 (1–3) [0–5] {2–2}	2 (1–3) [0–5] {2–2}	1 (0–1) [0–5] {1–1}
**Perceived predictability**	1 (0–3) [0–5] {1–2}	1 (0–3) [0–5] {1–2}	1 (0–2) [0–5] {1–3}

*Note:* Shown are medians, (IQR), [range] and {95% CIs} and *n* (%) as appropriate. For symptoms during a flare, these were scored using Numerical Rating Scale (NRS) from 0 = no symptoms, to 10 = worst symptoms imaginable. For burden shown are *n* (%) who scored ‘some’ or ‘a lot’ (out of ‘none’, ‘only a little’, ‘some’ ‘a lot’) for each of ‘how much do your symptoms keep you from doing the kinds of things you would usually do?’, ‘how much do you think about your symptoms’ and ‘how much do your symptom flares bother you?’ for each length flare. Scoring for flare self‐efficacy (‘how confident are you that you can cope and engage with daily activities, despite the flare?’) and perceived predictability (‘how predictable do you feel your flares are?’) were for both 0 and 5 (with 0 = not at all, and 5 = completely confident/entirely predictable). For descriptions of ‘other’ see Table S1.

Abbreviations: A&E, accident and emergency/emergency room/urgent care; HCP, healthcare provider.

### 3.3 Activities Undertaken During a Flare

The vast majority did not contact a healthcare provider in any way during a flare: for short flares, *n* = 81 (91%); for medium flares, *n* = 121 (91%), and for long flares, *n* = 166 (82%). When asked why they did not, 5 themes were identified.

#### 3.3.1 Theme 1: What Can They Do?

This theme is underpinned by a sense of futility about seeking help. It incorporated the subthemes: ‘healthcare providers don't help’, ‘healthcare providers can't help’ and ‘healthcare providers tell me what I already know or give me what I already have’.

Doctors don't seem to know anything about endometriosis or tell me that there's nothing they can do.

There is never any change in advice. I have done this in the past, and they advise my painkillers or things I already do i.e., hot water bottle. There is never any new advice so I have stopped asking for it.

#### 3.3.2 Theme 2: I Can Cope, It Will End

This theme encompasses the feeling that symptoms will pass without seeking help. Subthemes included: ‘I can cope by myself’, ‘it will end’, ‘it is not bad enough’, ‘only contact if bad or long enough’.

I've got prescription medications to manage the pain and I'm used to dealing with the ebbs and flows of chronic disease.

Because I will only go if the pain reaches a 10, I can handle a 9 just about.

#### 3.3.3 Theme 3: Broken Healthcare System

This theme describes barriers to seeking help that are related to a healthcare system under pressure. Subthemes included ‘burden on healthcare systems’ as well as healthcare being ‘inaccessible with long wait’.

‘I feel like I'm bothering them … and putting more strain on NHS [healthcare services]’.

Because I would spend my life trying to talk to someone.

#### 3.3.4 Theme 4: Perceived Dismissal and Gaslighting

This theme includes previous negative experiences of healthcare, including worries about being judged or dismissed by healthcare providers

I have C‐PTSD from Medical Professionals. Invalidating my pain and gaslighting me. For example: I complained of rectal bleeding during my period. I was asked if I am wiping the right hole.

The emotional toll of not being heard isn't worth the effort.

#### 3.3.5 Theme 5: Symptoms Prevent Me Seeking Help

This theme comprises responses around symptoms during flares being a barrier to contacting healthcare providers. For example, the symptoms were too intense or too frequent.

Times I've been in so much pain I've been unable to leave my house to seek help.

I would be there every day!

### 3.4 Identified Triggers for Flares

52.8% (*n* = 124) reported being able to identify triggers for their flares, with *n* = 111 (47.2%) reporting not being able to identify triggers.

**FIGURE 1:**
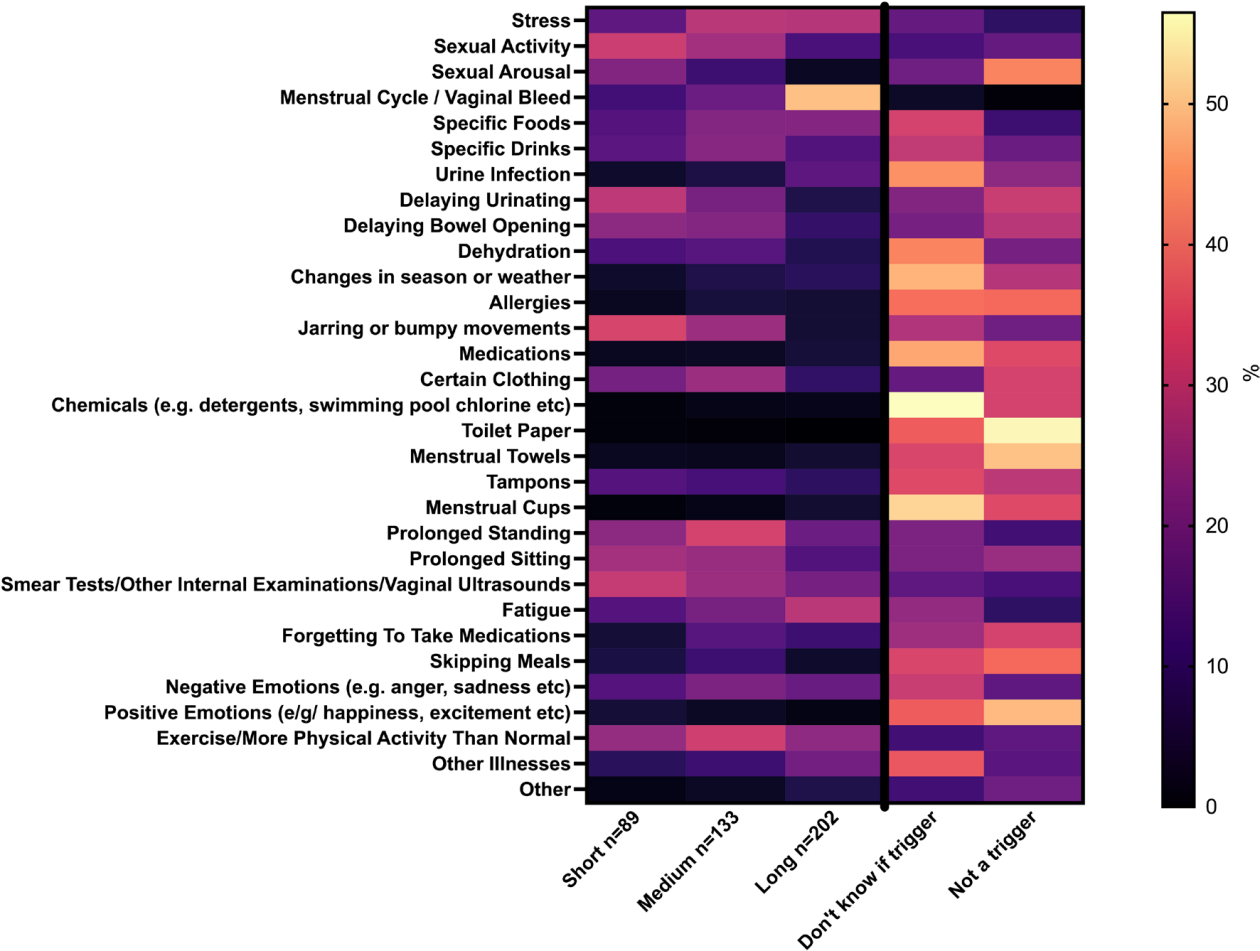
Triggers for flares. Shown are the percentages of participants reporting each potential trigger (lighter colour is higher percentage), with percentages are calculated based on the number of people reporting that length of flare (numbers shown on *x* axis for difference length flares). Percentages for ‘don't know if trigger’ and ‘not a trigger’ are shown out of *n* = 124 who reported identifying triggers for their flares. Some participants selected prefer not to say: *N* = 3 for ‘sexual activity’, *n* = 2 for ‘sexual arousal’, *n* = 1 for ‘menstrual cycle’, *n* = 1 for ‘specific drinks’, *n* = 1 for ‘urine infection’, *n* = 1 for ‘changes in season/weather’, *n* = 1 for ‘medications’, *n* = 1 for ‘prolonged sitting’, *n* = 1 for ‘forgetting to take medications’, *n* = 1 for ‘skipping meals’, *n* = 1 for ‘positive emotions’ and *n* = 2 for ‘other’.

Figure 1 shows all potential triggers. Most common trigger for long flares was the menstrual cycle, with fatigue and stress also being reported. For medium flares, prolonged standing and exercise/more physical activity than normal were both reported frequently. For short flares, sexual activity, internal examinations (smear tests/vaginal ultrasounds) and bumpy movements were all reported triggers.

### 3.5 Impact of Flares: Self‐Efficacy, Predictability, Burden and Quality of Life

Table 2 shows the predictability, flare self‐efficacy and burden of each flare length. Flares of all lengths had a marked burden on daily life, keeping from usual activities, thinking about flares and being bothersome. Many participants reported that they are ‘not at all’ confident coping when experiencing flares (for short flares *n* = 16 (18.6%), for medium flares *n* = 26 (20.6%) and for long flares *n* = 62 (31.2%)). Additionally, around a third of participants found their flares were ‘not at all’ predictable (for short flares *n* = 29 (33.7%), for medium flares *n* = 46 (36.2%) and for long flares *n* = 55 (27.6%)).

Across all flare lengths, we found significant negative correlations between flare self‐efficacy and burden (for short flares *n* = 86: thinking about symptoms rho = −0.336, *p* = 0.012, stopping usual activities rho = −0.572, *p* < 0.001, bothersomeness rho = −0.425, *p* < 0.001; for medium flares *n* = 124: thinking about rho = −0.512, *p* < 0.001, stopping usual activities rho = −0.752, *p* < 0.001, bothersomeness rho = −0.497, *p* < 0.001; for long flares *n* = 199: thinking about rho = −0.364, *p* < 0.001, stopping usual activities rho = −0.507, *p* < 0.001, bothersomeness rho = −0.327, *p* < 0.001). We found no significant correlation between perceived predictability and burden for any length flare.

When exploring whether the perceived predictability of flares, or flare self‐efficacy, correlated with quality‐of‐life we found for long flares there was a significant correlation between flare self‐efficacy and EHP‐30 Pain subscale (rho = −0.35, *p* < 0.001), meaning greater flare self‐efficacy was associated with lower impact of pain on quality‐of‐life; and between flare self‐efficacy and EHP‐30 Control & Powerlessness (rho = −0.249, *p* < 0.001), meaning greater flare self‐efficacy was associated with lower scores for powerlessness. We did not find any significant correlations after correction with other flare lengths or with perceived predictability.

We also explored the estimated total time spent in flares, with a median of 96 h per month spent in flare (IQR 48–285.6), {CIs 80.363–149}. This was significantly correlated with quality‐of‐life measures: EHP‐30 Pain rho = 0.344, *p* < 0.001 and EHP‐30 Control & Powerlessness rho = 0.211, *p* = 0.035.

### 3.6 Treatments and Strategies for Managing Flares

**FIGURE 2:**
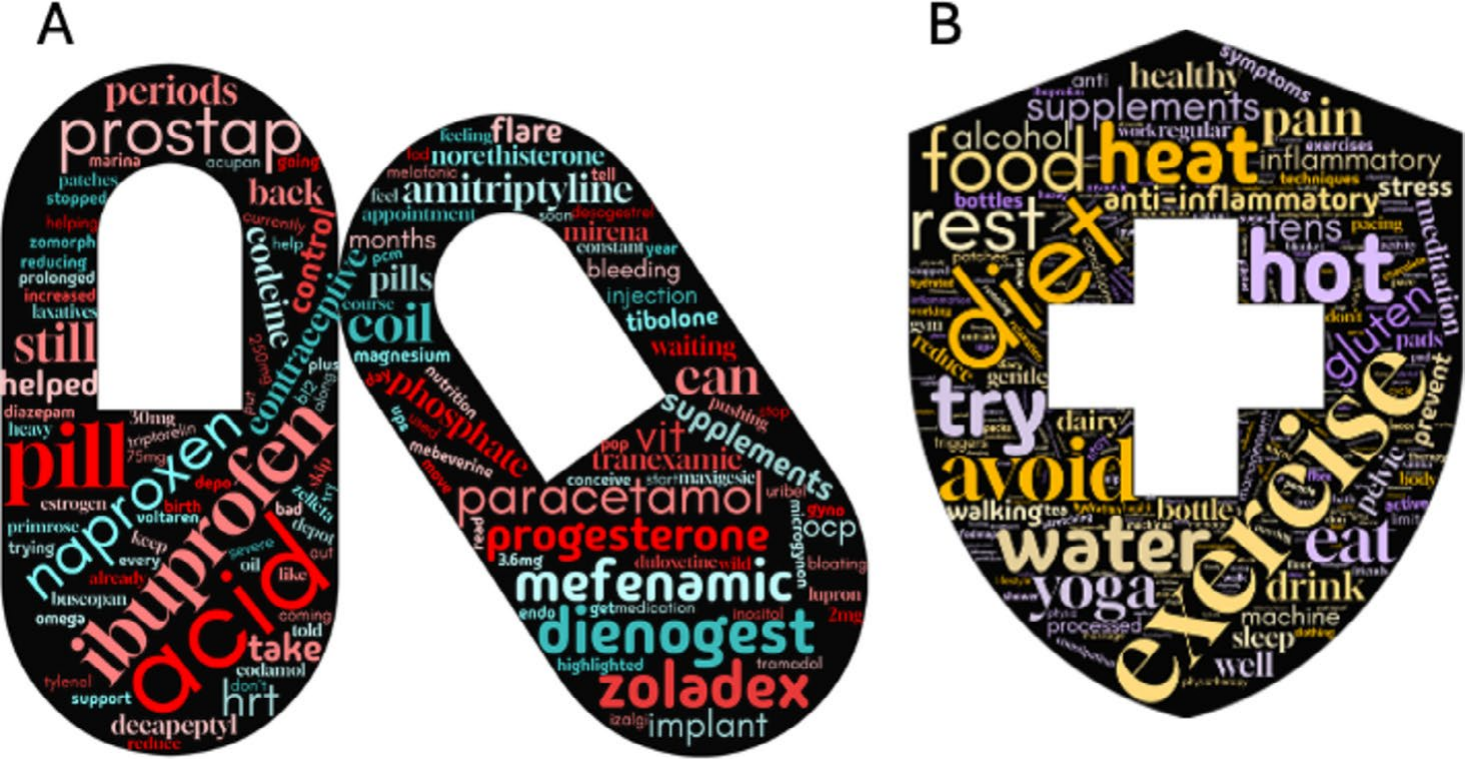
Wordclouds created from responses to treatments and strategies used to prevent flares. (A) Shows medications, and (B) Shows other strategies or activities used. The size of the words reflect the number of responses which included them. These are descriptive visual summaries to highlight the lack of consensus and conflicting methods (e.g., exercise and rest).

*n* = 60 (25.8%) of participants reported taking medication to prevent flares. This included hormonal medications, vitamins/supplements and analgesics (see Figure 2A). Overall, participants reported this as working relatively well, with a median of 4 (IQR 0–5) [range −10 to +10] {CIs 2–4}.

*n* = 110 (47.6%) reported using other strategies or activities to prevent flares, including dietary strategies, exercise, pacing and rest (see Figure 2B for summary of all strategies/activities). These strategies were again overall reported as quite helpful, median 3 (IQR 2–5) [range −1 to +10] {CIs 3–4}.

Most participants reported taking medication to treat flares as they occur *n* = 163 (71%), with these often being analgesics, ranging from over‐the‐counter NSAIDs to prescribed opiates. These were also reported as working relatively well, median 4 (IQR 2–5) [range –7 to +10] {CIs 3–4}.

### 3.7 Advice on Flares: Who From and How Helpful

Of our participants, *n* = 82 (35.3%) reported having received advice from a healthcare provider about how to manage flares. When asked whether this advice was helpful (0 = not at all, 1 = only a little, 2 = some, 3 = a lot), the median was 1 (IQR 0–1) [range 0–3] {CIs 1–1}. Many participants reported medical advice, such as certain medications, as well as advice around strategies such as heat, pacing and rest. Some also reported specific physiotherapy exercises and using a TENS machine.

More participants, *n* = 99 (42.7%) reported having received/found advice on flares from support groups or social media. This was reported as median 2 (IQR 2–3) [range 0–3] {CIs 2–2} for how helpful this was. This advice was generally more holistic, with many reports of diet, heat, rest, supplements and yoga. Many also added that social media/support groups helped with feelings of isolation.

For those that reported advice from both healthcare provider and social media/support groups, we compared how helpful this advice was, and a significant difference was found (*Z* = −4.679, *p* < 0.001), with social media/support groups being reported as more helpful.

## 4 Discussion

### 4.1 Main Findings

This study highlights that it is extremely common for those with endometriosis to experience symptom flares, particularly long flares, with participants spending a median of 96 h in flare per month. These flares have a large impact on daily life and increased hours spent in flare per month were associated with lower quality‐of‐life. Although approximately half of participants could identify triggers, flares were generally experienced as unpredictable. A wide variety of medication and self‐management strategies are described, and these are often helpful. However, participants reported low self‐efficacy with regards to their flares. The majority (over 80%) do not contact healthcare providers. Reasons for not seeking help could be categorised into 5 themes: ‘what can they do?’, ‘I can cope, it will end’, ‘broken healthcare system’, ‘perceived dismissal and gaslighting’ and ‘symptoms stop me’. Critically, many have not received advice from healthcare providers about flares, and obtain information from support groups/social media instead of, or as well as, professional advice. Interestingly, those who reported advice from both sources often considered advice from support groups/social media to be more useful. This study was conducted using patient support sites and online recruitment, meaning that our cohort is likely to consist of individuals with the highest symptom burden, and it cannot be assumed to represent all individuals with endometriosis.

### 4.2 Strengths & Limitations

This is the largest study using the modified MAPP Flares questionnaire to characterise flares in endometriosis‐associated pain and other symptoms. As such it provides us with new information regarding triggers for flares and associated health seeking and self‐management strategies during flares. Importantly, combining these data with qualitative responses allows greater insights into the reasons for not engaging with healthcare providers and paints a bleak picture of the participants' previous experiences. The inclusion of measures of predictability and self‐efficacy informed by wider work in chronic pain highlights the importance of these measures and strengthens the clinical utility of our findings.

However, there are limitations to this work. The use of online recruitment and data collection whilst facilitating recruitment of a more geographically diverse sample limits the study to only those with access to the internet. Whilst recruitment was online, the questions were only available in English and therefore there is a language bias to our findings which future work should address. Importantly, recruiting via support groups potentially biases our sample to those whose symptoms are most impactful, who do experience flares, and who utilise support groups for information. Alongside this, there is a non‐response bias in that those who do not experience flares are less likely to complete the survey; thus, this cannot be used for prevalence estimates. Although our qualitative survey data allows us to develop themes to understand barriers to treatment, future in‐depth qualitative research, facilitating dialogue, would deepen this understanding. Additionally, participants are asked to report on previous experiences and symptoms during flares, which, like all research in pain, is susceptible to perception bias.

As our study is about symptomatology, we have not captured data on stage of disease and purposely included those with a working diagnosis of endometriosis, as well as those with surgically confirmed disease. Whilst this means that potentially some of our participants may not have endometriosis lesions, we felt this reflected current varied thinking on the need for a diagnosis for good clinical management and includes the voices of those with a working diagnosis that are frequently excluded from research in EAP, helping to reduce diagnostic access bias. Additionally, we aimed to minimise questionnaire burden for participants and therefore only collected limited information where it did not address a key aim of the study. Thus, with our limited demographic data we are unable to determine how representative the sample is in relation to race, sexuality or gender.

### 4.3 Interpretation

In line with our previous work, flares clearly impact on QoL, however, here we show that individuals are often not receiving advice about flares from healthcare providers. This is perhaps not surprising given that there are no published clinical trials of pharmacological or other approaches (e.g., psychological, lifestyle, physiotherapy) in the prevention or treatment of symptom flares for those with endometriosis and no discussion of prevention, management or treatment of flares in clinical guidelines on endometriosis [7, 8]. Notably, many participants report similar strategies/medications for both prevention of and treatment during a flare. With the data available we are unable to know if this is because experience suggests these approaches are useful in both situations, whether these are used as these are the only resources accessible, or if our questions were not interpreted as we expected. Nonetheless, our study highlights prevention and treatment of symptom flares as an area of unmet clinical need, and the authors hope to see more research into this area in the future.

It is important to note that despite low flare self‐efficacy our participants do not routinely seek help from healthcare providers during flares. Whilst we would advocate self‐management strategies, and it is unrealistic to expect healthcare to be accessible as frequently as many report having flares, the reasons given for not accessing help are concerning. In the majority these themes are negative and informed by past experiences. Disappointingly the theme of ‘perceived dismissal and gaslighting’ mirrors other experiences of endometriosis‐related care [21, 22], but here, it emphasises the long‐term impact of negative experiences as barriers to accessing future care. We cannot know whether the theme of ‘broken healthcare systems’ would have been present previously. However, it is clear that the care of those with endometriosis was and continues to be impacted by the COVID‐19 pandemic and ongoing challenges within the NHS in the UK [23, 24]. One theme which stands out is ‘what can they do?’, the view that healthcare providers are not able to help. Further research is needed to explore whether healthcare providers feel similarly that they do not have the skills and/or tools to help during flares, particularly given that clinical guidelines do not help with this. However, even if healthcare providers are readily available, our findings show that for many the severity of symptoms limits their ability to seek help (‘symptoms stop me’). This highlights the need for strategies that can be implemented at home. On a positive note, the theme ‘I can cope, it will end’ reflects the temporality of flares and flare self‐efficacy, meaning that help from healthcare providers is not always felt necessary.

Given the low flare self‐efficacy we observe and the correlation with both the burden of flares and quality‐of‐life domains (specifically the EHP‐30 Pain and Control & Powerlessness subscales), improving flare self‐efficacy may be an important target for treatment. Methods integral to pain management programmes, including cognitive behavioural therapy and exercise, have been evidenced to increase pain self‐efficacy in patients with chronic low back pain [25, 26]. In addition, advice and guidance for managing flares is commonly discussed during a pain management programme with the aim of ensuring patients feel better equipped to cope with flares when they occur [27, 28, 29]. However, there is currently no evidence in endometriosis on how to improve flare self‐efficacy, or the impact improving flare self‐efficacy might have on quality of life or flare burden.

Furthermore, health anxiety and worry about symptoms is common for people who experience persistent symptoms [30]. It is important to acknowledge that it can be difficult for patients to confidently discern what might be an exacerbation of an existing chronic condition (such as in a flare) from an acute problem occurring in addition to their chronic condition. Interventions which include education on pain mechanisms, and which aim to help patients build confidence in understanding their pain experiences are likely to increase recognition of pain flares, associated triggers and engagement with flare management strategies.

## 5 Conclusion

This study explores flares in symptoms in people with endometriosis‐associated pain, an area which has received little research focus despite its clinical importance. Our findings highlight the burden of symptom flares and the lack of confidence patients have in their own and their healthcare providers' ability to manage flares. Further research is urgently needed to better understand the mechanisms underlying flares and develop an evidence base for personalised strategies for preventing, managing and treating them. It will be important that the perspectives of both patients and health care professionals are considered in future work in this area to ensure interventions, if proven to be effective, can successfully be integrated into both clinical practice and self‐management advice.

## Author Contributions

L.C.: conception, planning, carrying out, analysing and writing up. E.E.: conception, planning, analysing and writing up. F.T.: conception, analysing and writing up. E.C.: conception, planning, carrying out, writing up. K.V.: conception, planning, carrying out, analysing and writing up.

## Funding

This study was approved by the Medical Sciences Interdivisional Research Ethics Committee, University of Oxford, R91127/RE001 (29.02.2024). L.C. is funded by the Medical Research Foundation as part of the UKRI Strategic Priorities Fund (SPF) Advanced Pain Discovery Platform (APDP). E.E. receives funding for research activities from the NIHR Oxford BRC.

## Consent

Implied consent was attained via tick boxes to ensure the participant was aged 18 or over and that they agreed to take part in the study.

## Conflicts of Interest

L.C. is a trustee of the Pelvic Pain Support Network. K.V. is a member of the medical advisory panel for Endometriosis UK. K.V. declares research funding from UKRI, NIHR, NIH US and honoraria for consultancy and talks and associated travel expenses paid to her institution from Gedeon Richter, Gesynta, Reckitts and Eli Lilly. E.E. is a member of the medical advisory panel for Endometriosis UK, and the NICE guideline committee for Endometriosis. F.T. has no disclosures. E.C. is CEO of Endometriosis UK.

## Supporting information


**Data S1:** bjo70211‐sup‐0001‐DataS1.pdf.

**Table S1:** Descriptions of ‘other’ responses.

## Data Availability

The data that support the findings of this study are available from the corresponding author upon reasonable request.
