# Supplementary material for: Symptom Flares in Endometriosis: Burden, Self‐Management and Barriers to Care in a Cross‐Sectional Survey
Source: BJOG. 2026 Mar 13;133(9):1731–41. doi: 10.1111/1471-0528.70211 (PMC13418943; doi:10.1111/1471-0528.70211)
Supplement: Supplementary file 1 — Data S1: bjo70211‐sup‐0001‐DataS1.pdf. [file BJO-133-1731-s001.pdf]

Endometriosis-associated pain - experience of 'flares'

Page 1

General Information

This study aims to investigate endometriosis-associated pain, particularly 'flares' where symptoms are much worse than usual.

We appreciate your interest in participating in this questionnaire/online survey. We are looking for participants with endometriosis who are 18 years of age or older. Please read through this information before agreeing to participate (if you wish to) by ticking the ‘yes’ box below.

You may ask any questions before taking part by contacting the researcher (details below).

We (the Nuffield Department of Women’s & Reproductive Health at the University of Oxford) are investigating endometriosis-associated pain.

You will be given some questions to read and then answer. It should take about 15 minutes. No background knowledge is required.

Do I have to take part?

Please note that your participation is voluntary. You may withdraw at any point during the questionnaire for any reason, before submitting your answers, by pressing the 'exit' button or closing the browser. After you have completed the survey your responses cannot be withdrawn (as responses are anonymous).

How will your data be used?

We will not ask for any data that could directly identify you. Although every reasonable effort has been taken, confidentiality during actual internet communication procedures cannot be guaranteed. Data may be stored on backups or server logs beyond the timeframe of this research project. It is not possible for us to access information about your IP address.

Your data will be stored in a password-protected file and may be used in academic publications. Most questions are optional. Research data will be stored for 5 years after publication or public release.

Who will have access to your data?

The data you provide may be shared with other researchers in the research group, under the direct supervision of the listed researchers (Dr Lydia Coxon and Prof Katy Vincent), to carry out analysis.

We would like to use the data in future studies, and to share data with other researchers (e.g. in online databases). Data will have identifying information removed before it is shared with other researchers or results are made public.

Responsible members of the University of Oxford may be given access to data for monitoring and/or audit of the study to ensure we are complying with guidelines, or as otherwise required by law.

The principal researcher is Dr Lydia Coxon, who is a researcher in the Department of Women's and Reproductive Health at the University of Oxford. This project is being completed with guidance from Prof Katy Vincent, of the same department.

This project has been reviewed by, and received ethics clearance through, the University of Oxford Central University Research Ethics Committee.

What is there is a problem?

If you have a concern about any aspect of this research, please contact Lydia Coxon (lydia.coxon@wrh.ox.ac.uk), and I will do my best to answer your query. I will acknowledge your concern within 10 working days and give you an indication of how it will be dealt with. If you remain unhappy or wish to make a formal complaint, please contact the Chair of the Research Ethics Committee at the University of Oxford who will seek to resolve the matter as soon as possible:

Medical Sciences Interdivisional Research Ethics Committee; Email: ethics@medsci.ox.ac.uk; Address: Research Services, University of Oxford, Boundary Brook House, Churchill Drive, Headington, Oxford OX3 7GB

Please note that you may only participate in this survey if you are 18 years of age or over.

PIS for Questionnaire v1 28/02/2024

Please tick this box if you are over the age of 18. ✱ *Required*

- ○ I certify that I am 18 years of age or older

If you have read the information about and agree to participant with the understanding that the data you submit will be processed accordingly, please tick the box below to get started. ✱ *Required*

- ○ Yes, I agree to take part.

Page 2

How old are you currently (years)?

Please enter a whole number (integer).

[ ]

Has there been a time in your life when you typically had pelvic pain during your periods (including irregular bleeding or bleeding while on hormonal treatments, but not spotting)?

- No pain
- Mild cramps (medication never or rarely needed)
- Moderate cramps (medication usually needed)
- Severe cramps (medication and bed rest needed)
- I would prefer not to say

Please rate how severe your pelvic pain during your period was **at its worst** in the last 12 months using a scale from 0 to 10 where 0=no pain and 10=worst imaginable pain.

Please don't select more than 1 answer(s) per row.

	0	1	2	3	4	5	6	7	8	9	10	
no pain	☐	☐	☐	☐	☐	☐	☐	☐	☐	☐	☐	worst imaginable pain

Please rate how severe your pelvic pain during your period was **on average** in the last 12 months using a scale from 0 to 10 where 0=no pain and 10=worst imaginable pain.

Please don't select more than 1 answer(s) per row.

	0	1	2	3	4	5	6	7	8	9	10	
no pain	☐	☐	☐	☐	☐	☐	☐	☐	☐	☐	☐	worst imaginable pain

Have you ever had pelvic pain during intercourse or in the 24 hours following vaginal sexual intercourse/penetration?

- No
- Yes
- I have not had vaginal sexual intercourse/penetration
- I would prefer not to say

Please rate how severe your pelvic pain during intercourse or in the 24 hours following vaginal sexual intercourse/penetration was **at its worst** in the last 12 months using a scale from 0 to 10 where 0=no pain and 10=worst imaginable pain.

Please don't select more than 1 answer(s) per row.

	0	1	2	3	4	5	6	7	8	9	10	

no pain	☐	☐	☐	☐	☐	☐	☐	☐	☐	☐	☐	worst imaginable pain

Please rate how severe your pelvic pain during intercourse or in the 24 hours following vaginal sexual intercourse/penetration was **on average** in the last 12 months using a scale from 0 to 10 where 0=no pain and 10=worst imaginable pain.

Please don't select more than 1 answer(s) per row.

	0	1	2	3	4	5	6	7	8	9	10	
no pain	☐	☐	☐	☐	☐	☐	☐	☐	☐	☐	☐	worst imaginable pain

Have you ever had pelvic pain in general? By 'pelvic pain in general' we mean any type of pain (cramping, shooting, stabbing etc) in the lower part of your belly. Please do not count: pain related to periods or intercourse, pregnancy or childbirth, any surgery, sports-related or other injury, food poisoning or stomach flu.

- ○ No
- ○ Yes
- ○ I would prefer not to say

Please rate how severe your pelvic pain in general was **at its worst** in the last 3 months using a scale from 0 to 10 where 0=no pain and 10=worst imaginable pain.

Please don't select more than 1 answer(s) per row.

	0	1	2	3	4	5	6	7	8	9	10	
no pain	☐	☐	☐	☐	☐	☐	☐	☐	☐	☐	☐	worst imaginable pain

Please rate how severe your pelvic pain in general was **on average** in the last 3 months using a scale from 0 to 10 where 0=no pain and 10=worst imaginable pain.

Please don't select more than 1 answer(s) per row.

	0	1	2	3	4	5	6	7	8	9	10	
no pain	☐	☐	☐	☐	☐	☐	☐	☐	☐	☐	☐	worst imaginable pain

How many years have you experienced pelvic pain of any kind (relating to your period, to vaginal intercourse/penetration, or in general)? Please give the number of years you have experienced any pelvic pain. *For example if you have experienced pain with vaginal penetration for 3 years, pelvic pain in general for 6 years and pain with periods for 10 years, please answer 10 years.*

Please enter a number.

____

Page 3

Have you been diagnosed with endometriosis?

- ☐ Yes, by laparoscopy/surgery
- ☐ Yes, by imaging (MRI or ultrasound)
- ☐ Yes, I have a working diagnosis, awaiting tests
- ☐ Yes, I have a working diagnosis, I am not awaiting tests
- ☐ No

What stage endometriosis do you have, if you have been told? Please note you can select more than one option.

- ☐ 1 (I)
- ☐ 2 (II)
- ☐ 3 (III)
- ☐ 4 (IV)
- ☐ Mild
- ☐ Moderate
- ☐ Severe
- ☐ Superficial
- ☐ Deep
- ☐ I am not sure / don't remember
- ☐ I haven't been told a stage

What medication do you take regularly (or on prescription) for your endometriosis / chronic pelvic pain?

- ☐ Combined hormonal contraceptives (the pill)
- ☐ Progesterone-only hormonal contraceptives (the mini-pill)
- ☐ Hormonal IUD (intrauterine device) (e.g. mirena coil)
- ☐ HRT (hormone replacement therapy)
- ☐ Menopause injections (GnRH analogues e.g. Zoladex, Prostap etc)
- ☐ Other hormonal medication
- ☐ Other IUD (intrauterine device) (e.g. copper coil)
- ☐ Paracetamol
- ☐ Iburprofen
- ☐ Aspirin
- ☐ Co-codomol or other codeine medication
- ☐ Duloxetine
- ☐ Pregabalin
- ☐ Gabapentin
- ☐ Amitriptyline
- ☐ Tramadol
- ☐ Oramorph
- ☐ Fentanyl patches
- ☐ Other

If you selected Other, please specify:

Where are you located?

- England
- Wales
- Scotland
- Northern Ireland
- Outside of the UK

Page 4

Have you ever had a flare of your endometriosis symptoms that lasted **less than an hour**? By flare we mean, symptoms that are much worse than usual? ✱ *Required*

- ◯ Yes
- ◯ No

On average, how many minutes do your flares usually last if they last **less than an hour**? Please give a number of minutes.

Please enter a number.

Please make sure the number is between 0 and 60.

How often do you usually have flares that last **less than an hour**? (Please use any time frame that applies to you, e.g. times per day, per week, per month etc.; please do not use terms such as several or few, but instead try to use a number (e.g. three times per day)).

How would you rate your symptoms during a typical flare that lasts **less than an hour**?

Please don't select more than 1 answer(s) per row.

	0 (no symptoms)	1	2	3	4	5	6	7	8	9	10 (worst symptoms)
Pain, pressure and discomfort associated with your pelvis and/or bladder	☐	☐	☐	☐	☐	☐	☐	☐	☐	☐	☐
Urgency to urinate (go for a wee)	☐	☐	☐	☐	☐	☐	☐	☐	☐	☐	☐
Urgency to open bowels (go for a poo)	☐	☐	☐	☐	☐	☐	☐	☐	☐	☐	☐
Frequency of urinating	☐	☐	☐	☐	☐	☐	☐	☐	☐	☐	☐
Frequency of pooing	☐	☐	☐	☐	☐	☐	☐	☐	☐	☐	☐
Overall bladder or pelvic pain symptoms	☐	☐	☐	☐	☐	☐	☐	☐	☐	☐	☐
Overall pain symptoms that were not bladder or pelvic (e.g. upper back, headache, etc)	☐	☐	☐	☐	☐	☐	☐	☐	☐	☐	☐

During a typical flare that lasts **less than an hour**, what is your single most bothersome symptom? (Please select only *one* answer)

- Pain, pressure or discomfort in your bladder
- Pain, pressure or discomfort in your vagina
- Pain, pressure or discomfort on the skin around the entrance to the vagina and/or back passage
- Pain, pressure or discomfort elsewhere in your pelvis
- Pain or discomfort during or after sexual activity
- Strong need to urinate with little or no warning
- Strong need to open your bowels with little or no warning
- Frequent urination during the day
- Frequent urination at night
- Frequent bowel opening during the day
- Frequent bowel opening at night
- Sense of not emptying your bladder completely
- Sense of not emptying your bowels completely
- Increased vaginal discharge
- A feeling of bloating in the abdomen
- Other

If you selected Other, please specify:

During a typical flare that lasts **less than an hour**, which of the following activities do you usually do? (Please check all that apply)

- Contact a health care provider (e.g. physician/doctor, nurse, physiotherapist etc) by telephone or email
- Go to see a health care provider
- Go to A&E/Emergency room or urgent care centre
- Have a medication changed (new medication or different dose)
- Rest
- Take medication used specifically to treat the flare
- Other

If you selected Other, please specify:

If you do not contact a health care provider by telephone or email; go to see a health care provider; or go to A&E/Emergency room or urgent care centre, please can you tell us why not?

If you take medication specifically to treat a flare, please can you give more information about what you take?

During a typical flare that lasts **less than an hour**, how much do your symptoms keep you from doing the kinds of things you would usually do?

- None
- Only a little
- Some
- A lot

During a typical flare that lasts **less than an hour**, how much do you think about your symptoms?

- None
- Only a little
- Some
- A lot

How much do your symptom flares that last **less than an hour** bother you?

- None
- Only a little
- Some
- A lot

How confident are you that you can cope and engage with daily activities, despite the flare that last **less than an hour**?

Please don't select more than 1 answer(s) per row.

	0	1	2	3	4	5	
Not at all confident	☐	☐	☐	☐	☐	☐	Completely confident

How predictable do you feel you pain flares that last **less than an hour** are?

Please don't select more than 1 answer(s) per row.

	0	1	2	3	4	5	
Not at all predictable	☐	☐	☐	☐	☐	☐	Entirely predictable

Page 5

Have you ever had a flare of your endometriosis symptoms that lasted **more than an hour but less than a day**? By flare we mean, symptoms that are much worse than usual? ✱ *Required*

- ◌ Yes
- ◌ No

On average, how many hours do your flares usually last if they last **more than an hour but less than a day**? Please give a number of hours.

Please enter a number.

Please make sure the number is between 1 and 24.

[ ]

How often do you usually have flares that last **more than an hour but less than a day**? (Please use any time frame that applies to you, e.g. times per day, per week, per month etc.; please do not use terms such as several or few, but instead try to use a number (e.g. three times per day)).

[ ]

How would you rate your symptoms during a typical flare that lasts **more than an hour but less than a day**?

Please don't select more than 1 answer(s) per row.

	0 (no symptoms)	1	2	3	4	5	6	7	8	9	10 (worst symptoms)
Pain, pressure and discomfort associated with your pelvis and/or bladder	☐	☐	☐	☐	☐	☐	☐	☐	☐	☐	☐
Urgency to urinate (go for a wee)	☐	☐	☐	☐	☐	☐	☐	☐	☐	☐	☐
Urgency to open bowels (go for a poo)	☐	☐	☐	☐	☐	☐	☐	☐	☐	☐	☐
Frequency of urinating	☐	☐	☐	☐	☐	☐	☐	☐	☐	☐	☐
Frequency of pooing	☐	☐	☐	☐	☐	☐	☐	☐	☐	☐	☐
Overall bladder or pelvic pain symptoms	☐	☐	☐	☐	☐	☐	☐	☐	☐	☐	☐
Overall pain symptoms that were not bladder or pelvic (e.g. upper back, headache, etc)	☐	☐	☐	☐	☐	☐	☐	☐	☐	☐	☐

During a typical flare that lasts **more than an hour but less than a day**, what is your single most bothersome symptom? (Please select only *one*

answer)

- Pain, pressure or discomfort in your bladder
- Pain, pressure or discomfort in your vagina
- Pain, pressure or discomfort on the skin around the entrance to the vagina and/or back passage
- Pain, pressure or discomfort elsewhere in your pelvis
- Pain or discomfort during or after sexual activity
- Strong need to urinate with little or no warning
- Strong need to open your bowels with little or no warning
- Frequent urination during the day
- Frequent urination at night
- Frequent bowel opening during the day
- Frequent bowel opening at night
- Sense of not emptying your bladder completely
- Sense of not emptying your bowels completely
- Increased vaginal discharge
- A feeling of bloating in the abdomen
- Other

If you selected Other, please specify:

During a typical flare that lasts **more than an hour but less than a day**, which of the following activities do you usually do? (Please check all that apply)

- Contact a health care provider (e.g. physician/doctor, nurse, physiotherapist etc) by telephone or email
- Go to see a health care provider
- Go to A&E/Emergency room or urgent care centre
- Have a medication changed (new medication or different dose)
- Rest
- Take medication used specifically to treat the flare
- Other

If you selected Other, please specify:

If you do not contact a health care provider by telephone or email; go to see a health care provider; or go to A&E/Emergency room or urgent care centre, please can you tell us why not?

If you take medication specifically to treat a flare, please can you give more information about what you take?

During a typical flare that lasts **more than an hour but less than a day**, how much do your symptoms keep you from doing the kinds of things you would usually do?

- None
- Only a little
- Some
- A lot

During a typical flare that lasts **more than an hour but less than a day**, how much do you think about your symptoms?

- None
- Only a little
- Some
- A lot

How much do your symptom flares that last **more than an hour but less than a day** bother you?

- None
- Only a little
- Some
- A lot

How confident are you that you can cope and engage with daily activities, despite the flare that last **more than an hour but less than a day**?

Please don't select more than 1 answer(s) per row.

	0	1	2	3	4	5	
Not at all confident	☐	☐	☐	☐	☐	☐	Completely confident

How predictable do you feel you pain flares that last **more than an hour but less than a day** are?

Please don't select more than 1 answer(s) per row.

	0	1	2	3	4	5	
Not at all predictable	☐	☐	☐	☐	☐	☐	Entirely predictable

Page 6

Have you ever had a flare of your endometriosis symptoms that lasted **more than a day**? By flare we mean, symptoms that are much worse than usual? ✱ *Required*

- ○ Yes
- ○ No

On average, how many days do your flares usually last if they last **more than a day**? Please give a number of days.

Please enter a number.

[ ]

How often do you usually have flares that last **more than a day**? (Please use any time frame that applies to you, e.g. times per day, per week, per month etc.; please do not use terms such as several or few, but instead try to use a number (e.g. three times per day)).

[ ]

How would you rate your symptoms during a typical flare that lasts **more than a day**?

Please don't select more than 1 answer(s) per row.

	0 (no symptoms)	1	2	3	4	5	6	7	8	9	10 (worst symptoms)
Pain, pressure and discomfort associated with your pelvis and/or bladder	☐	☐	☐	☐	☐	☐	☐	☐	☐	☐	☐
Urgency to urinate (go for a wee)	☐	☐	☐	☐	☐	☐	☐	☐	☐	☐	☐
Urgency to open bowels (go for a poo)	☐	☐	☐	☐	☐	☐	☐	☐	☐	☐	☐
Frequency of urinating	☐	☐	☐	☐	☐	☐	☐	☐	☐	☐	☐
Frequency of pooing	☐	☐	☐	☐	☐	☐	☐	☐	☐	☐	☐
Overall bladder or pelvic pain symptoms	☐	☐	☐	☐	☐	☐	☐	☐	☐	☐	☐
Overall pain symptoms that were not bladder or pelvic (e.g. upper back, headache, etc)	☐	☐	☐	☐	☐	☐	☐	☐	☐	☐	☐

During a typical flare that lasts **more than a day**, what is your single most bothersome symptom? (Please select only *one* answer)

- Pain, pressure or discomfort in your bladder
- Pain, pressure or discomfort in your vagina
- Pain, pressure or discomfort on the skin around the entrance to the vagina and/or back passage
- Pain, pressure or discomfort elsewhere in your pelvis
- Pain or discomfort during or after sexual activity
- Strong need to urinate with little or no warning
- Strong need to open your bowels with little or no warning
- Frequent urination during the day
- Frequent urination at night
- Frequent bowel opening during the day
- Frequent bowel opening at night
- Sense of not emptying your bladder completely
- Sense of not emptying your bowels completely
- Increased vaginal discharge
- A feeling of bloating in the abdomen
- Other

If you selected Other, please specify:

During a typical flare that lasts **more than a day**, which of the following activities do you usually do? (Please check all that apply)

- ☐ Contact a health care provider (e.g. physician/doctor, nurse, physiotherapist etc) by telephone or email
- ☐ Go to see a health care provider
- ☐ Go to A&E/Emergency room or urgent care centre
- ☐ Have a medication changed (new medication or different dose)
- ☐ Rest
- ☐ Take medication used specifically to treat the flare
- ☐ Other

If you selected Other, please specify:

If you do not contact a health care provider by telephone or email; go to see a health care provider; or go to A&E/Emergency room or urgent care centre, please can you tell us why not?

If you take medication specifically to treat a flare, please can you give more information about what you take?

During a typical flare that lasts **more than a day**, how much do your symptoms keep you from doing the kinds of things you would usually do?

- None
- Only a little
- Some
- A lot

During a typical flare that lasts **more than a day**, how much do you think about your symptoms?

- None
- Only a little
- Some
- A lot

How much do your symptom flares that last **more than a day** bother you?

- None
- Only a little
- Some
- A lot

How confident are you that you can cope and engage with daily activities, despite the flare that last **more than a day**?

Please don't select more than 1 answer(s) per row.

	0	1	2	3	4	5	
Not at all confident	☐	☐	☐	☐	☐	☐	Completely confident

How predictable do you feel you pain flares that last **more than a day** are?

Please don't select more than 1 answer(s) per row.

	0	1	2	3	4	5	
Not at all predictable	☐	☐	☐	☐	☐	☐	Entirely predictable

Page 7

Have you identified any triggers for your flares?

- ○ Yes
- ○ No

In the table below, please tick any of the factors that you think trigger a flare of each duration. For each potential trigger you can also select 'don't know if a trigger', 'not a trigger' or 'prefer not to say'.

Please don't select more than 3 answer(s) per row.

	less than an hour	more than an hour but less than a day	more than a day	don't know if a trigger	not a trigger	prefer not to say
Stress	☐	☐	☐	☐	☐	☐
Sexual activity	☐	☐	☐	☐	☐	☐
Sexual arousal	☐	☐	☐	☐	☐	☐
Menstrual cycle/vagina bleed	☐	☐	☐	☐	☐	☐
Specific foods	☐	☐	☐	☐	☐	☐
Specific drinks	☐	☐	☐	☐	☐	☐
Urine infection	☐	☐	☐	☐	☐	☐
Delaying urinating	☐	☐	☐	☐	☐	☐
Delaying bowel opening	☐	☐	☐	☐	☐	☐
Dehydration	☐	☐	☐	☐	☐	☐
Changes in season or weather	☐	☐	☐	☐	☐	☐
Allergies	☐	☐	☐	☐	☐	☐
Jarring or bumpy movements	☐	☐	☐	☐	☐	☐
Medications	☐	☐	☐	☐	☐	☐
Certain clothing	☐	☐	☐	☐	☐	☐
Chemicals (e.g. detergents, swimming pool chlorine etc)	☐	☐	☐	☐	☐	☐
Toilet paper	☐	☐	☐	☐	☐	☐
Sanitary towels	☐	☐	☐	☐	☐	☐
Tampons	☐	☐	☐	☐	☐	☐
Menstrual cups	☐	☐	☐	☐	☐	☐
Prolonged standing	☐	☐	☐	☐	☐	☐
Prolonged sitting	☐	☐	☐	☐	☐	☐
Smear tests/other internal examinations/vaginal ultrasounds	☐	☐	☐	☐	☐	☐
Fatigue	☐	☐	☐	☐	☐	☐
Forgetting to take medication	☐	☐	☐	☐	☐	☐
Skipping meals	☐	☐	☐	☐	☐	☐
Negative emotions e.g. anger, sadness etc	☐	☐	☐	☐	☐	☐
Positive emotions e.g. happiness, excitement etc	☐	☐	☐	☐	☐	☐
Exercise/more physical activity than normal	☐	☐	☐	☐	☐	☐
Other illnesses	☐	☐	☐	☐	☐	☐
Other: please describe below	☐	☐	☐	☐	☐	☐

If you selected 'Other' please describe here:

Page 8

Do you take any medications to prevent flares?

- Yes
- No

If you selected Yes, please tell us what medication this is:

How well do you feel this medication prevents flares in your symptoms? Please score from -10 to 10, where 0 = no change at all, +10 = my symptoms went away completely, -10 = my symptoms became very much worse.

Please don't select more than 1 answer(s) per row.

	-10	-9	-8	-7	-6	-5	-4	-3	-2	-1	0 (no change)	+1
Completely worse	☐	☐	☐	☐	☐	☐	☐	☐	☐	☐	☐	☐

Do you use any other strategies or activities to prevent flares?

- Yes
- No

If you selected Yes, please tell us what strategy or activity this is:

How well do you feel this strategy or activity prevents flares in your symptoms? Please score from -10 to 10, where 0 = no change at all, +10 = my symptoms went away completely, -10 = my symptoms became very much worse.

Please don't select more than 1 answer(s) per row.

	-10	-9	-8	-7	-6	-5	-4	-3	-2	-1	0 (no change)	+1
Completely worse	☐	☐	☐	☐	☐	☐	☐	☐	☐	☐	☐	☐

Do you take any medications to treat flares as they occur?

- Yes
- No

If you selected Yes, please tell us what medication this is:

How well do you feel this medication treats flares in your symptoms? Please score from -10 to 10, where 0 = no change at all, +10 = my symptoms went away completely, -10 = my symptoms became very much worse.

Please don't select more than 1 answer(s) per row.

	-10	-9	-8	-7	-6	-5	-4	-3	-2	-1	0 (no change)	+1
Completely worse	☐	☐	☐	☐	☐	☐	☐	☐	☐	☐	☐	☐

Have you received advice from a health care provider (doctor, nurse, physiotherapist, psychologist) about how to manage flares?

- ○ Yes
- ○ No

Did you find this advice helpful?

- ○ Not at all
- ○ Only a little
- ○ Some
- ○ A lot

Please give us more information about the advice you were given from a healthcare provider:

Have you received / found advice on flares from support groups or social media?

- ☐ Yes
- ☐ No

Did you find this advice helpful?

- ○ Not at all
- ○ Only a little
- ○ Some
- ○ A lot

Please give us more information about the advice you received via support groups / social media

If there is anything you would like to tell us about 'flares' that we have not already asked, please put it here.

Page 9

During the last 4 weeks, how often because of your endometriosis have you...

Please don't select more than 1 answer(s) per row.

	Never	Rarely	Sometimes	Often	Always
Been unable to go to social events because of the pain?	☐	☐	☐	☐	☐
Been unable to do jobs around the home because of the pain?	☐	☐	☐	☐	☐
Found it difficult to stand because of the pain?	☐	☐	☐	☐	☐
Found it difficult to sit because of the pain?	☐	☐	☐	☐	☐
Found it difficult to walk because of the pain?	☐	☐	☐	☐	☐
Found it difficult to exercise or do the leisure activities you would like to do because of the pain?	☐	☐	☐	☐	☐
Lost your appetite and/or been unable to eat because of the pain?	☐	☐	☐	☐	☐
Been unable to sleep properly because of the pain?	☐	☐	☐	☐	☐
Had to go to bed/lie down because of the pain?	☐	☐	☐	☐	☐
Been unable to do the things you want to do because of the pain?	☐	☐	☐	☐	☐
Felt unable to cope with the pain?	☐	☐	☐	☐	☐
Generally felt unwell?	☐	☐	☐	☐	☐
Felt frustrated because your symptoms are not getting better?	☐	☐	☐	☐	☐
Felt frustrated because you are not able to control your symptoms?	☐	☐	☐	☐	☐
Felt unable to forget your symptoms?	☐	☐	☐	☐	☐
Felt as though your symptoms are ruling your life?	☐	☐	☐	☐	☐
Felt your symptoms are taking away your life?	☐	☐	☐	☐	☐
Felt depressed?	☐	☐	☐	☐	☐
Felt weepy/tearful?	☐	☐	☐	☐	☐
Felt miserable?	☐	☐	☐	☐	☐
Had mood swings?	☐	☐	☐	☐	☐
Felt bad tempered or short tempered?	☐	☐	☐	☐	☐
Felt violent or aggressive?	☐	☐	☐	☐	☐
Felt unable to tell people how you feel?	☐	☐	☐	☐	☐
Felt others do not understand what you are going through?	☐	☐	☐	☐	☐
Felt as though others think you are moaning?	☐	☐	☐	☐	☐

Felt alone?	☐	☐	☐	☐	☐
Felt frustrated as you cannot always wear the clothes you would choose?	☐	☐	☐	☐	☐
Felt your appearance has been affected?	☐	☐	☐	☐	☐
Lacked confidence?	☐	☐	☐	☐	☐

Section A: These questions concern the effect endometriosis has had on your work during the last 4 weeks. If you have not been in paid or voluntary employment during the last 4 weeks please tick here and move onto Section B

○ I have not been I paid or voluntary employment during the last 4 weeks

During the last 4 weeks, how often because of your endometriosis have you...

Please don't select more than 1 answer(s) per row.

	Never	Rarely	Sometimes	Often	Always
Had to take time off work because of the pain?	☐	☐	☐	☐	☐
Been unable to carry out duties at work because of the pain?	☐	☐	☐	☐	☐
Felt embarrassed about symptoms at work?	☐	☐	☐	☐	☐
Felt guilty about taking time off work?	☐	☐	☐	☐	☐
Felt worried about not being able to do your job?	☐	☐	☐	☐	☐

Section B: These questions concern the effect endometriosis has had on your relationship with your child/children during the last 4 weeks. If you do not have any children please tick here and move onto Section C.

○ I do not have any children

During the last 4 weeks, how often because of your endometriosis have you...

Please don't select more than 1 answer(s) per row.

	Never	Rarely	Sometimes	Often	Always
Found it difficult to look after your child/children?	☐	☐	☐	☐	☐
Been unable to play with your child/children?	☐	☐	☐	☐	☐

Section C: These questions concern the effect endometriosis has had on your sexual relationships during the last 4 weeks.

How often during the last 4 weeks because of your endometriosis have you...

Please don't select more than 1 answer(s) per row.

	Never	Rarely	Sometimes	Often	Always	If not relevant please tick here
Experienced pain during or after intercourse?	☐	☐	☐	☐	☐	☐
Felt worries about having intercourse because of the pain?	☐	☐	☐	☐	☐	☐
Avoided intercourse because of the pain?	☐	☐	☐	☐	☐	☐
Felt guilty about not wanting to have intercourse?	☐	☐	☐	☐	☐	☐
Felt frustrated because you cannot enjoy intercourse?	☐	☐	☐	☐	☐	☐

Section D: These questions concern your feelings during the last 4 weeks about the medical profession. If this section is not relevant for you please tick here and move on to Section E.

○ Not relevant for me

During the last 4 weeks, how often because of your endometriosis have you...

Please don't select more than 1 answer(s) per row.

	Never	Rarely	Sometimes	Often	Always
Felt the doctor(s) you have seen is (are) not doing anything for you?	☐	☐	☐	☐	☐
Felt the doctor(s) think it is all in your mind?	☐	☐	☐	☐	☐
Felt frustrated at the doctor(s) lack of knowledge about endometriosis?	☐	☐	☐	☐	☐
Felt like you are wasting the doctor(s) time?	☐	☐	☐	☐	☐

Section E: These questions concern your feelings during the last 4 weeks about your treatment for endometriosis. Treatment means any surgery or prescribed medication for your endometriosis. If this question is not relevant to you please tick here and move onto Section F.

○ This is not relevant to me.

During the last 4 weeks, how often because of your endometriosis have you...

Please don't select more than 1 answer(s) per row.

	Never	Rarely	Sometimes	Often	Always
Felt frustrated because treatment is not working?	☐	☐	☐	☐	☐
Found it difficult coping with the side effects of treatment?	☐	☐	☐	☐	☐
Felt annoyed at the amount of treatment you have had to have?	☐	☐	☐	☐	☐

Section F: These questions concern your problems conceiving during the last 4 weeks. If this section is not relevant to you please tick here and move onto the next page.

○ This is not relevant to me.

During the last 4 weeks, how often because of your endometriosis have you...

Please don't select more than 1 answer(s) per row.

	Never	Rarely	Sometimes	Often	Always
Felt worried about the possibility of not having children/more children?	☐	☐	☐	☐	☐
Felt inadequate because you may not/have not been able to have children/more children?	☐	☐	☐	☐	☐
Felt depressed at the possibility of not having children/more children?	☐	☐	☐	☐	☐
Felt that the possibility of not conceiving/not being able to conceive had put a strain upon your personal relationship?	☐	☐	☐	☐	☐

Page 10: Final page

Thank you very much for completing this survey. We very much appreciate the time you have taken.

If you have any questions, please feel free to contact one of the researchers Lydia Coxon (lydia.coxon@wrh.ox.ac.uk).
