# Supplementary material for: Symptom Flares in Endometriosis: Burden, Self‐Management and Barriers to Care in a Cross‐Sectional Survey
Source: BJOG. 2026 Mar 13;133(9):1731–41. doi: 10.1111/1471-0528.70211 (PMC13418943; doi:10.1111/1471-0528.70211)
Supplement: Supplementary file 2 — Table S1: Descriptions of ‘other’ responses. [file BJO-133-1731-s002.docx]

*Supplementary Table 1:* ***Descriptions of ‘other’ responses.*** *Shown are n(%) and text. For full list of activities during a flare and single most bothersome symptom during a flare, see Table 2.*

|  | **Short** | **Medium** | **Long** |
| --- | --- | --- | --- |
| **Activities during flare, n (%)** | | | |
| Other | 11 (12.360): heat/hot water bottle (n=5); squat/lie down on side/ curl up (n=4); jovi ‘nanotech’; solpadeine max; co-codamol; drink water | 9 (6.767):  Yoga / gentle exercise (n=3); heat/hot water bottle (n=4); dietary changes; meditate (n=2); paracetamol / ibuprofen (n=2); bath; pelvic physio exercises | 32 (15.842):  paracetamol/ ibuprofen/ codeine (n=2); heat/hot water bottle (n=14); ice pack/ cooling mask (n=2); TENS; castor oil (n=2); bath (n=4); bed rest; yoga; French – “burn myself to have another pain”; herbal pain patches; drink lots; pressure/massage (n=2); essential oil; solpadeine max; sitting/crouching/ lie down/curl up (n=2); co-codamol; take A/L; exercise more |
| **Single most bothersome symptom during flare** | | | |
| Other | 3 (3.371):  Pain in legs; pain in rectum; painful lung collapse and shortness of breath | 5 (3.759):  All of the above and shooting pains left side, back and legs; chest pain and shortness of breath; pain and cramping in groin, pelvis and lower abdomen; pain in leg, back and left ovary; pain on right side and across belly | 20 (9.901):  All of the above (n=2); bowel and bladder obstruction; lower back and pelvic pain (n=2); deep pain in mid-section; tiredness not relieved by sleeping; headache; migraine and lower back pain; leg and lower back pain (n=2); cramping and pain lower abdomen, groin and pelvis; whole body pain and tingling sensation; nausea and dizziness; nausea, numbness in leg, inability to sleep and facial swelling; pain in legs and arms; pain on right side lower back and stomach; pain with digestion; exhaustion and full body pain; vomiting, nausea and burning pain. |
|  |  |  |  |
